# Peripheral Blood CD4/CD8 Ratio Predicts Out‐of‐Specification Products in Lisocabtagene Maraleucel Manufacturing

**DOI:** 10.1002/cnr2.70413

**Published:** 2025-11-27

**Authors:** Taichi Hirano, Hiro Tatetsu, Shikiko Ueno, Takafumi Shichijo, Takahisa Nakamura, Asami Yamada, Mitsuhiro Uchiba, Koji Kato, Kisato Nosaka, Masao Matsuoka, Jun‐ichirou Yasunaga

**Affiliations:** ^1^ Department of Haematology, Rheumatology, and Infectious Diseases Kumamoto University Hospital Kumamoto Japan; ^2^ Department of Medicine and Biosystemic Science Kyushu University Graduate School of Medicine Fukuoka Japan

**Keywords:** CD4, CD8, chimeric antigen receptor T‐cell, out‐of‐specification

## Abstract

**Background:**

Lisocabtagene maraleucel (liso‐cel) is a CD19‐directed chimeric antigen receptor T‐cell therapy for patients with relapsed or refractory large B‐cell lymphoma.

**Methods:**

In this retrospective study involving 22 patients, we evaluated the association between peripheral blood CD4/CD8 ratios and the occurrence of out‐of‐specification (OOS) products during manufacturing. Expanded access protocols have permitted the infusion of OOS products, despite their failure to meet commercial release criteria, under close regulatory oversight.

**Results:**

Five patients who received OOS products had significantly lower CD4/CD8 ratios (median 0.22 vs. 0.46; *p* = 0.008).

**Conclusion:**

Our findings suggest that a low CD4/CD8 ratio (below one‐third) may be a predictive marker for OOS outcomes, potentially supporting patient selection and treatment planning. Therefore, prospective validation using a larger cohort is warranted.

## 1 Introduction

Chimeric antigen receptor T‐cell (CAR‐T‐cell) therapy has transformed the treatment landscape for patients with relapsed or refractory large B‐cell lymphoma (LBCL), offering unprecedented outcomes in the salvage setting [1, 2, 3]. A crucial aspect of CAR T‐cell therapy is the manufacturing process, which can result in one of three main outcomes: (1) a compliant product that meets all predefined specifications; (2) a manufacturing failure in which no final product is produced; or (3) an out‐of‐specification (OOS) product, in which CAR‐T cells are generated but fail to meet one or more release criteria (e.g., cell count or viability) [4]. From a regulatory perspective, any deviation from the required manufacturing standards, whether due to laboratory results or documentation issues, is considered a nonconforming product (United States Food and Drug Administration Biologics License Application 125714) [5].

Despite the high success rates, CAR‐T cell manufacturing failures are reported in 1%–13% of cases, often due to inadequate T cell expansion [1, 2, 3]. Although OOS products do not meet commercial standards, they can be administered under expanded access protocols and provide life‐saving options for patients who have exhausted conventional therapies. However, these products carry risks due to their reduced viability or altered T cell composition, which can reduce efficacy and increase toxicity. In addition, the regulatory reviews required for OOS product approval can prolong vein‐to‐vein time (V2Vt), potentially delaying treatment, which may result in poor outcomes [6].

Lisocabtagene maraleucel (liso‐cel) is a CD19‐directed CAR‐T cell therapy approved for patients with relapsed or refractory LBCL [3]. Worel et al. recently reported OOS rates of 1%–6% for CD19‐directed CAR T‐cell products, including 5% for liso‐cel [7]. In contrast, our cohort showed a higher OOS frequency (> 20%), which provided the rationale for conducting the present study. Although the risk factors associated with OOS outcomes during manufacturing remain poorly understood, the prediction of OOS may aid in patient selection and timely apheresis. This study aimed to identify potential predictors of OOS during liso‐cel manufacturing, with a focus on the peripheral blood CD4/CD8 ratio before leukapheresis.

## 2 Methods

### 2.1 Patients

We retrospectively reviewed all consecutive patients (*N* = 22) who underwent leukapheresis for liso‐cell manufacturing at Kumamoto University Hospital between May 2022 and June 2024. Written informed consent was obtained from each patient before the procedure. This study was approved by the Institutional Review Board (No. 499) of Kumamoto University Hospital and conducted in accordance with the principles of the Declaration of Helsinki.

### 2.2 Apheresis Procedure

Mononuclear cells were collected via continuous mononuclear cell collection using the Spectra Optia system (Terumo BCT). Blood flow was adjusted to the maximum tolerated continuous flow rate (up to 100 mL/min). Anticoagulation was achieved using acid citrate dextrose solution A at a ratio of 1:15 (adjusted to 1:10–1:15 case for platelet aggregation). The processed target blood volume was either 7000 or 12 000 mL, depending on the protocol.

### 2.3 Clinical Biomarker

Clinical data of patients included clinical characteristics and pre‐apheresis hematological parameters. CD4^+^ or CD8^+^ T cell counts in the peripheral blood were quantified using an AQUIOS flow cytometry system (Beckman Coulter Inc.).

### 2.4 Definition of Manufacturing Failure and OOS

Manufacturing failure was defined as the inability to produce a CAR‐T cell product that met the required dose or viability threshold. OOS products were defined as those that did not meet the commercial release criteria but were still considered suitable for infusion under expanded access protocols. Among the 22 patients, one manufacturing failure and five OOS cases were identified. Specific details of the OOS findings are not available due to confidentiality agreements. To focus on the risk factors associated with OOS, the manufacturing failure case was excluded from this analysis.

### 2.5 Statistical Analysis

The Fisher's exact test was used to compare categorical variables between the OOS and compliant groups. The Mann–Whitney *U* test was used for continuous variables with a non‐parametric distribution. Specificity/sensitivity and receiver operating characteristic (ROC) curves were generated to assess the utility and thresholds of the potential indicators. Thresholds were calculated using the Youden index. All the statistical analyses were performed using EZR version 1.64 (based on R version 4.3.1; Vienna, Austria). Statistical significance was set at *p* < 0.05.

## 3 Results

### 3.1 Baseline Characteristics and Apheresis Parameters

Among the 22 patients who underwent leukapheresis for liso‐cel manufacturing, 14 patients had diffuse large B‐cell lymphoma, one patient had primary mediastinal large B‐cell lymphoma, and seven patients had transformed follicular lymphoma. OOS products were observed in three patients with diffuse large B‐cell lymphoma and in two patients with transformed follicular lymphoma. The manufacturing failure occurred in one patient with diffuse large B‐cell lymphoma. We compared the baseline demographics and peripheral blood parameters at the time of leukapheresis between the OOS (*n* = 5) and compliant (in‐spec) groups (*n* = 16) (Table 1).

**TABLE 1:** Comparison of baseline characteristics and apheresis parameters.

	Out of spec	In‐spec	SD or SMD	*p*
Number of patients	5	16		
Sex, Male *n* (%)	2/5 (40%)	7/16 (43.8%)	0.076^a^	1.00^c^
Age, Median (range)	63 (56–68)	67.5 (28–76)	0.072^b^	0.301^d^
Body weight kg, Median (range)	59 (47.8–67.6)	55.65 (42.6–86.8)	0.127^b^	0.934^d^
Prior HSCT, *n* (%)	2/5 (40%)	3/16 (18.8%)	0.480^a^	0.553^c^
Previous bendamustine Tx, *n* (%)	1/5 (20%)	5/16 (31.2%)	0.260^a^	1.00^c^
ALC ×10^6^/L, Median (range)	1369 (910–1480)	735 (280–2330)	1.122^b^	0.017^d^
CD4 ×10^6^/L, Median (range)	130 (77–199)	165 (79–477)	0.714^b^	0.248^d^
CD8 ×10^6^/L, Median (range)	676 (325–1056)	337.5 (89–1271)	1.031^b^	0.026^d^
CD4/CD8 ratio, Median (range)	0.22 (0.10–0.31)	0.46 (0.17–1.64)	1.299^b^	0.008^d^
Hematocrit %, Median (range)	29.7 (28.1–33.1)	31.1 (24.4–36.8)	0.244^b^	0.563^d^
Platelet ×10^10^/L, Median (range)	14.7 (7.9–16.7)	15.85 (4.3–22.7)	0.040^b^	0.650^d^
Apheresis product volume mL, median (range)	286 (237–317)	298 (226–350)	0.457^b^	0.457^d^
Blood volume, processed 12 000 mL, *n* (%)	4/5 (80%)	14/16 (87.5%)	0.204^a^	1.00^c^

Abbreviations: ALC, absolute lymphocyte counts; HSCT, hematopoietic stem cell transplantation.

^a^ Standerdized difference.

^b^ Standerdized mean difference.

^c^ Fisher's exact test.

^d^ Mann–Whitney *U* test.

**FIGURE 1:**
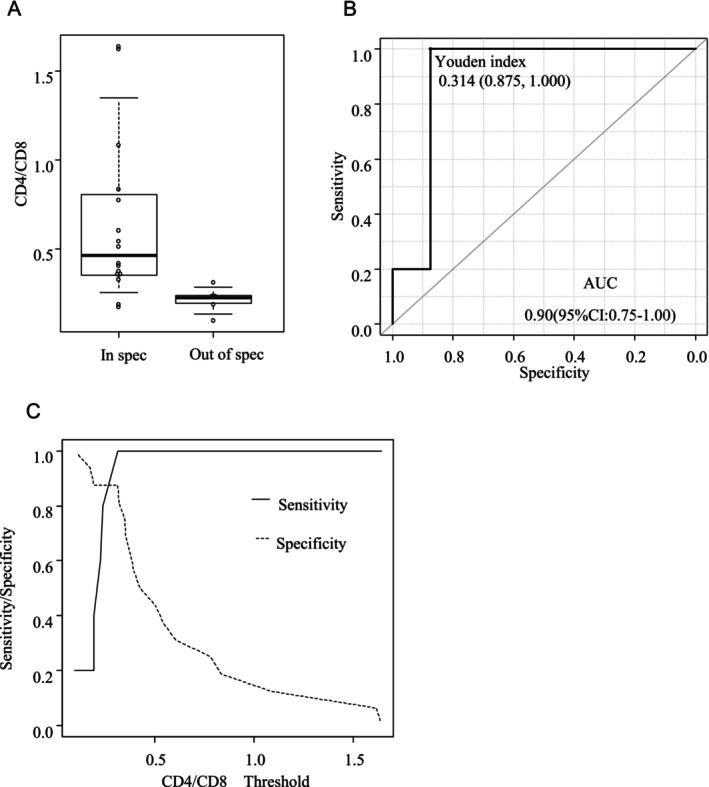
Peripheral blood CD4/CD8 ratio and its predictive value for generation of out‐of‐specification (OOS) products (A) Comparison of the peripheral blood CD4/CD8 ratio between patients with the generation of out‐of‐specification (OOS) (*n* = 5) and compliant (*n* = 16) products. The OOS group exhibited a significantly lower median ratio (*p* = 0.008). (B) Receiver operating characteristic (ROC) curve of the CD4/CD8 ratio for predicting the OOS status. The area under the curve (AUC) was 0.90 (95% confidence interval [95% CI]: 0.75–1.00), demonstrating excellent predictive performance. (C) Sensitivity and specificity for predicting the generation of OOS products at various CD4^+^/CD8^+^ ratio thresholds. A cutoff of one‐third provided an optimal balance.

No significant differences in sex distribution (40% female vs. 43.8% male; *p* = 1.00), age (median 63 vs. 67.5 years; *p* = 0.301), body weight, prior hematopoietic stem cell transplantation, or history of bendamustine exposure were noted between the OOS and compliant groups. Hematocrit levels, platelet counts, and apheresis collection volumes were comparable between the two groups. However, the lymphocyte‐related parameters showed significant differences. Compared with the compliant group, the OOS group demonstrated significantly higher absolute lymphocyte counts (ALC: median 1369 vs. 735 × 10^6^/L; *p* = 0.017) and CD8^+^ T‐cell counts (676 vs. 337.5 × 10^6^/L; *p* = 0.026). In contrast, CD4^+^ T‐cell counts were not significantly different (130 vs. 165 × 10^6^/L; *p* = 0.248). The CD4/CD8 ratio was significantly lower in the OOS group than in the compliant group (median 0.22 vs. 0.46; *p* = 0.008), with the largest standardized mean difference (SMD = 1.299) among all measured variables (Table 1, Figure 1A).

### 3.2 Predictive Utility of the CD4/CD8 Ratio for OOS Outcomes

Given the significant association between the CD4/CD8 ratio and OOS product generation, we further assessed its discriminative utility using ROC curve analysis (Figure 1B). The analysis revealed a high area under the curve (AUC) of 0.90 (95% confidence interval: 0.75–1.00), indicating excellent predictive performance.

To explore a practical threshold for clinical use, we examined the sensitivity and specificity across a range of CD4/CD8 cutoff values (Figure 1C). A threshold of one‐third provided a favorable balance, effectively identifying patients at an increased risk of OOS product generation. These findings suggest that a peripheral blood CD4/CD8 ratio below one‐third during leukapheresis may serve as a predictive biomarker for OOS risk in liso‐cel manufacturing.

## 4 Discussion

A recognized challenge in CAR T‐cell therapy is the risk of manufacturing failure or generation of OOS products, which can adversely affect the efficacy and timeliness of these treatments. Several factors have been implicated in CAR‐T manufacturing failure, including high concentrations of total nucleated cells, elevated CD3^+^ cell counts, and increased neutrophil counts in the apheresis product [8]. Additional risk factors, such as prior exposure to bendamustine, low platelet counts, and low CD4/CD8 ratios, have been reported [9]. Similarly, potential predictors specific to OOS status, including advanced disease stage, a high International Prognostic Index score, and older patient age, have been proposed [9]. However, the clinical outcomes of OOS products may approximate those of in‐specification products [10, 11, 12], suggesting that OOS designation does not necessarily imply reduced efficacy.

In this study, OOS products were associated with a lower CD4/CD8 ratio, higher ALC, and increased proportion of CD8^+^ T cells. This suggests that the presence of many effector CD8^+^ cells relative to CD4^+^ cells may interfere with cell growth and viability during manufacturing. These findings are consistent with those of previous reports, showing that an imbalance in T cell subsets can weaken the quality of CAR T cells [9]. Although the exact mechanisms remain unknown, a shift toward CD8^+^ predominance may reduce the long‐term efficacy of the final product.

Real‐world data, including recent findings from the United Kingdom National CAR‐T Panel, indicate that OOS products can serve as viable therapeutic options for certain patients [13, 14, 15, 16]. However, the additional regulatory reviews required to administer these products may prolong V2Vt, thereby delaying treatment. Therefore, it is crucial to balance prompt intervention with product quality. By identifying a low CD4/CD8 ratio (below one‐third) as a risk factor for OOS products, our study highlights the potential value of pre‐leukapheresis immune profiling. Avoiding T‐cell‐deteriorating chemotherapy and attempting tailored apheresis strategies on T cell subsets, particularly in patients at high risk, may help reduce OOS rates and shorten V2Vt.

This study had some limitations. The specific causes of OOS remain unclear because of confidentiality agreements, which limit mechanistic insights. Evaluation of further T‐cell components, exhaustion markers (e.g., TIM3, LAG3, CTLA4, PD1), and proliferation capacity will be warranted to assess T‐cell fitness. Our sample size was small to perform regression analysis, and the retrospective design may have introduced a selection bias. Larger prospective studies in more diverse populations are needed to confirm the robustness of the CD4/CD8 ratio as a predictive biomarker and to explore interventions for improving manufacturing consistency.

In conclusion, this study shows that a peripheral blood CD4/CD8 ratio below one‐third exhibits a clinically relevant association with increased risk of OOS products during liso‐cel manufacturing. This ratio is expected to serve as a surrogate marker of T‐cell fitness, potentially guiding decisions on the optimal timing for leukapheresis and modifications to bridging therapies. Further research is needed to confirm these findings and develop standardized approaches to minimize manufacturing errors and OOS in CAR‐T therapy.

## Author Contributions

**Taichi Hirano:** conceptualization (lead), data curation (lead), formal analysis (lead), investigation (lead), project administration (equal), resources (equal), visualization (lead), writing – original draft (equal), writing – review and editing (equal). **Hiro Tatetsu:** conceptualization (equal), data curation (equal), funding acquisition (lead), investigation (equal), resources (equal), supervision (lead), writing – original draft (equal), writing – review and editing (lead). **Shikiko Ueno:** resources (equal), writing – review and editing (equal). **Takafumi Shichijo:** investigation (equal), resources (equal), writing – review and editing (equal). **Takahisa Nakamura:** investigation (equal), writing – review and editing (equal). **Asami Yamada:** resources (equal), writing – review and editing (equal). **Mitsuhiro Uchiba:** resources (equal), writing – review and editing (equal). **Koji Kato:** supervision (equal), writing – review and editing (equal). **Kisato Nosaka:** supervision (equal), writing – review and editing (equal). **Masao Matsuoka:** supervision (equal), writing – review and editing (equal). **Jun‐ichirou Yasunaga:** supervision (equal), writing – review and editing (equal).

## Funding

This study was partially supported by a grant from the JSPS KAKENHI (Grant Number 24K11563).

## Ethics Statement

This study was approved by the Institutional Review Board of Kumamoto University Hospital (No. 499) and was conducted in accordance with the principles of the Declaration of Helsinki. Written informed consent was obtained from all patients.

## Conflicts of Interest

Hiro Tatetsu has received honoraria from Bristol Myers Squibb, AstraZeneca, Chugai Pharmaceutical, Eisai, Ono, Nihon Shinyaku, Novartis, SymBio Pharmaceuticals Limited, Takeda Pharmaceutical, Meiji Seika Pharma, Gilead Sciences, and AbbVie Inc., and patents and royalties from mesoblasts. S.U. received honoraria from Sobi Japan, Asahi Kasei Pharma Corporation, Alexion, Novartis Pharmaceuticals, and Chugai Pharmaceutical Co., Argenx, Japan. K.K. received honoraria from Novartis, Chugai, Meiji, Gilead Sciences, Kyowa Kirin, and Bristol‐Myers Squibb, and research funding from Chugai, Takeda, AbbVie, Novartis, Eisai, Janssen, Ono, Meiji, Daiichi Sankyo, MSD, Bristol‐Myers Squibb, Gilead Sciences, and Astellas. K.N. received honoraria from Bristol Myers Squibb; Chugai Pharmaceutical; Eisai; Meiji Seika Pharma; and AbbVie Inc. Minophagen, Dai‐ichi Sankyo, Kyowa Kirin, and research funding from Kyowa Kirin and Chugai Pharmaceutical. J.Y. received honoraria from Bristol Myers Squibb; Chugai Pharmaceutical, Gilead Sciences, and AbbVie Inc. The remaining authors declare no conflicts of interest. The authors declare that the pharmaceutical companies from which they received honoraria or research funding were not involved in the designing of the study; collection, analysis or interpretation of the data; writing of the manuscript; or in the decision to submit the manuscript for publication. The relationships that these companies had with the authors were limited to academic lecture fees and research grants, which were unrelated to the present study.

## Data Availability

Data inquiries should be directed to tatetsu@kumamoto-u.ac.jp. Data will be made available to achieve the aims of the approved proposal.

## References

[1] S. S. Neelapu , F. L. Locke , N. L. Bartlett , et al., “Axicabtagene Ciloleucel CAR T‐Cell Therapy in Refractory Large B‐Cell Lymphoma,” New England Journal of Medicine 377 (2017): 2531–2544, 10.1056/NEJMoa1707447. 29226797 PMC5882485

[2] S. J. Schuster , M. R. Bishop , C. S. Tam , et al., “Tisagenlecleucel in Adult Relapsed or Refractory Diffuse Large B‐Cell Lymphoma,” New England Journal of Medicine 380 (2019): 45–56, 10.1056/NEJMoa1804980. 30501490

[3] J. S. Abramson , M. L. Palomba , L. I. Gordon , et al., “Lisocabtagene Maraleucel for Patients With Relapsed or Refractory Large B‐Cell Lymphomas (TRANSCEND NHL 001): A Multicentre Seamless Design Study,” Lancet 396 (2020): 839–852, 10.1016/S0140-6736(20)31366-0. 32888407

[4] C. Baguet , J. Larghero , and M. Mebarki , “Early Predictive Factors of Failure in Autologous CAR T‐Cell Manufacturing and/or Efficacy in Hematologic Malignancies,” Blood Advances 8 (2024): 337–342, 10.1182/bloodadvances.2023011992. 38052048 PMC10788849

[5] US FDA , Biologics License Application 125714 – Breyanzi (Lisocabtagene Maraleucel) (FDA, 2021).

[6] S. Vadgama , M. C. Pasquini , R. T. Maziarz , et al., ““Don't Keep Me Waiting”: Estimating the Impact of Reduced Vein‐to‐Vein Time on Lifetime US 3L+ LBCL Patient Outcomes,” Blood Advances 8 (2024): 3519–3527, 10.1182/bloodadvances.2023012240. 38662645 PMC11261112

[7] N. Worel , J. E. Mooyaart , J. D. Hoogenboom , et al., “CAR‐T Cell Manufacturing Failures and Out‐of‐Specification Products in the Real‐World Setting: A Survey From the EBMT Cellular Therapy and Immunobiology Working Party,” Bone Marrow Transplantation 60 (2025): 1184–1186, 10.1038/s41409-025-02623-0. 40374897

[8] C. Guillaume , C. NGO‐Chin , N. Douki , et al., “Failure and out of Specification Manufacturing of Autologous CAR‐T Cells Could Be Associated With a High Concentration of Total Nucleated Cells, CD3^+^ Cells and Neutrophils in the Apheresis Product,” Blood 142, no. Supplement 1 (2023): 3520, 10.1182/blood-2023-174865.

[9] T. Jo , S. Yoshihara , Y. Okuyama , et al., “Risk Factors for CAR‐T Cell Manufacturing Failure Among DLBCL Patients: A Nationwide Survey in Japan,” British Journal of Haematology 202 (2023): 256–266, 10.1111/bjh.18831. 37096915

[10] V. Dulobdas , A. A. Kirkwood , F. Serpenti , et al., “Risk Factors for CD19‐Targeting CAR T Manufacturing Failure and Patient Outcomes: A Report From the UK National CAR T Panel,” Blood 142, no. Supplement 1 (2023): 495, 10.1182/blood-2023-178834. 40032870 PMC11876324

[11] E. A. Chong , B. L. Levine , and S. Schuster , “Clinical Outcomes for Anti‐CD19 CAR T Cell (CTL019) Products Not Meeting Commercial Release Specifications,” Cytotherapy 22, no. Supplement (2020): S29.

[12] K. Kato , J. Kato , H. Goto , et al., “Clinical Outcomes of Japanese Patients Treated With Out‐of‐Specification Tisagenlecleucel in a Phase 3b Trial,” Cytotherapy 27 (2025): 938–943, 10.1016/j.jcyt.2025.04.067. 40377509

[13] S. Fried , R. Shouval , N. Varda‐Bloom , et al., “Point‐of‐Care CAR T‐Cell Therapy as Salvage Strategy for Out‐of‐Specification Tisagenlecleucel,” Leukemia & Lymphoma 63 (2022): 3385–3393, 10.1080/10428194.2022.2123232. 36111694

[14] B. Bowden , D. Ciccone , J. Salmon , V. Alegria , L. Kallenbach , and K. C. de Braganca , “Using Real‐World Remanufacturing and Recollection Data to Optimize Time‐to‐Treatment in Patients With Out‐of‐Specification Ciltacabtagene Autoleucel,” Blood 142, no. Supplement 1 (2023): 6918, 10.1182/blood-2023-186163.

[15] V. Dulobdas , A. A. Kirkwood , F. Serpenti , et al., “Risk Factors for CAR T‐Cell Manufacturing Failure and Patient Outcomes in Large B‐Cell Lymphoma: A Report From the UK National CAR T Panel,” Blood Cancer Journal 15 (2025): 30, 10.1038/s41408-025-01225-9. 40032870 PMC11876324

[16] P. Caimi , S. J. Schuster , L. J. Nastoupil , et al., “Outcomes in Patients (pts) With R/R Large B‐Cell Lymphoma (LBCL) Who Received Nonconforming Product (NCP) to Commercial Release of Lisocabtagene Maraleucel (liso‐cel) in the United States (US),” Transplant Cellular Therapy 30, no. Supplement (2024): S188–S189, 10.1016/j.jtct.2023.12.244.

